# Vulvar Varicosities in an Adolescent Girl with Morbid Obesity: A Case Report

**DOI:** 10.3390/children8030202

**Published:** 2021-03-07

**Authors:** Aikaterini Giannouli, Vasiliki Rengina Tsinopoulou, Artemis Tsitsika, Efthimios Deligeoroglou, Flora Bacopoulou

**Affiliations:** 1 Center for Adolescent Medicine and UNESCO Chair on Adolescent Health Care, First Department of Pediatrics, School of Medicine, National and Kapodistrian University of Athens, Aghia Sophia Children’s Hospital, 11527 Athens, Greece; giannouli.katerina@gmail.com; 2 Unit of Paediatric Endocrinology and Metabolism, 2nd Department of Paediatrics, School of Medicine, Aristotle University of Thessaloniki, AHEPA University Hospital, 54621 Thessaloniki, Greece; vasotsino@gmail.com; 3 Adolescent Health Unit, 2nd Department of Pediatrics, School of Medicine, National and Kapodistrian University of Athens, “P. & A. Kyriakou” Children’s Hospital, 11527 Athens, Greece; info@youth-health.gr; 4 Department of Pediatric & Adolescent Gynecology, Mitera Children’s Hospital, 15123 Athens, Greece; deligeoroglou@yahoo.gr

**Keywords:** adolescence, vulvar, varicosities, vulva, obesity, varicose veins, pelvic venous disorder, pelvic congestion syndrome

## Abstract

Vulvar varicosities in nonpregnant females, either isolated or as a part of the pelvic congestion syndrome, are rare. We present a case of an adolescent girl with morbid obesity with bilateral bluish protrusions on the labia minora, as an incidental finding, that coincided with her excessive weight gain. The adolescent underwent thorough clinical examination, doppler ultrasound, contrast venography and varicography, and magnetic resonance angiography to rule out alternative diagnoses. Imaging results confirmed the presence of large venous lakes. Venous drainage to the internal iliac vein and connections with the long saphenous vein were delineated. Incompetence, dilatation, or reflux of ovarian or internal iliac veins, or their main tributaries, were not noted. Since the adolescent was asymptomatic and other pathologies, such as vascular malformations or hemangiomas were excluded, she was managed conservatively with counseling about lifestyle modification and weight reduction. This is only the third reported case of vulvar venous varicosities in adolescents. Female sex, along with obesity, are known risk factors for varicose vein formation; however, the pathogenesis is not fully understood. Additional research is needed to elucidate the role of excess adipose tissue in the pathophysiology of vulvar varicose veins and to optimize diagnostic workup and management in adolescence.

## 1. Introduction

Vascular varicosities, i.e., dilated venous channels in the vulvar area, are rare and almost exclusively affect women during pregnancy. Almost 4–22% of pregnant women present with vulvar varicosities. The majority of cases disappear either immediately after parturition or during puerperium, and only 4–8% persist or worsen with time [1,2,3]. The prevalence of the disorder in nonpregnant women is not estimated, because only a handful of cases are published in literature [4,5]. Vulvar varicosities are included in pelvic venous syndromes (PVS). PVS are disorders of the pelvic venous circulation and, in 24–34% of the cases, coincide with visible varicosities on the vulva, thigh, or gluteal area [2]. These disorders are developed by increased vascular pressure due to obstruction, compression, valvular incompetence, or both [1]. Obesity has a divergent effect on PVS; increasing weight is associated with a higher prevalence of leg varicosities and lower prevalence of proximal vein flow disorders [6,7,8].

Most females with visible vulvar varices do not report any symptoms, especially during pregnancy. Palpable mass on the external genitalia, vaginal discomfort, swelling, heaviness, and pain are rare manifestations. These symptoms usually deteriorate at the end of the day after standing up, exercise, or coitus. Due to the paucity of symptoms and the embarrassment to report changes in the external genitalia, most cases of vulvar varices in pregnant women are diagnosed at term or even during labor.

## 2. Case Presentation

A 16-year-old Caucasian girl was referred to the Center for Adolescent Medicine and UNESCO Chair on Adolescent Health Care of the First Department of Pediatrics, School of Medicine, National and Kapodistrian University of Athens, at the Aghia Sophia Children’s Hospital. Her main concerns were oligomenorrhea and a mass over her external genitalia observed by the referring gynecologist.

The adolescent presented with a history of excessive weight gain (over 30 kg during the past 2 years), recently diagnosed Hashimoto’s thyroiditis, and oligomenorrhea. She had a family history of type 2 diabetes mellitus. She had menarche at 12 years of age, and she reported normal menstrual cycles during the first two gynecological years. In the last two years, menses were irregular with secondary amenorrhea for 4 months. She denied recent trauma at the vulvar or perineal area, and her mother reported that no vulvar mass had been observed in her past pediatric health visits. The adolescent did not complain of heaviness, discomfort, or regional pain at any position or following exercise or prolonged standing. She did not report sexarche; however, a urine pregnancy test was performed, and pregnancy was ruled out. 

**Figure 1:**
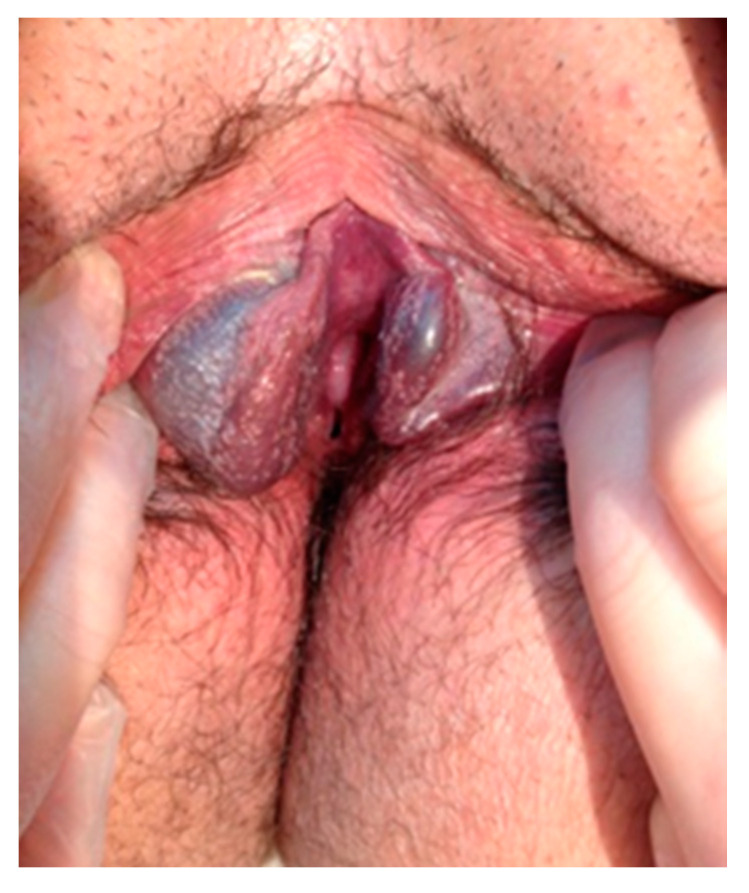
Vulvar varicosities observed in the lithotomy position.

Initially, the girl was examined in the lithotomy position and then in the upright position. In the lithotomy position, bilateral bluish protrusions were observed on labia minora (Figure 1). On the right side, the size of the mass was 3 cm × 1.5 cm, and on the left, there was a smaller mass of 1.5 cm × 1 cm. Both masses were soft, nontender, and variable in size with palpation. With the Valsalva maneuver and while standing, the masses enlarged, but they reduced in size with pressure. The perineum, the inguinal area, the gluteal region, and the lower limbs were free of varices. The external genitalia were otherwise normal. 

The adolescent had morbid obesity, with a body mass index of 41.9 Kg/m^2^ (WHO Class III obesity); however, her arterial blood pressure (124/74 mmHg) was below the hypertensive range for her age and height [9]. The development of her sexual characteristics was complete (Tanner stage V for breast and pubic hair), and she had hirsutism, acanthosis nigricans over the axillary areas, as well as abdominal striae. She had no palpable abdominal or pelvic masses. 

**Figure 2:**
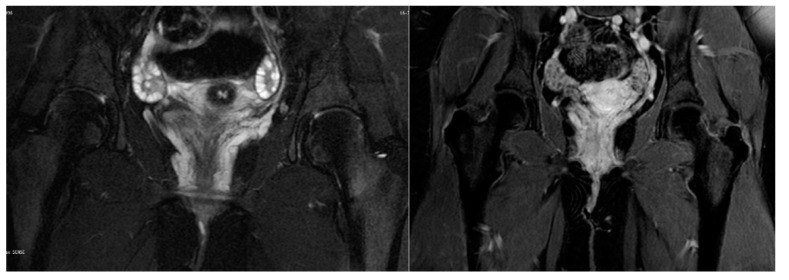
Magnetic resonance angiography: Vulvar varices draining into dilated pelvic floor tributaries of internal iliac vein.

Ultrasound evaluation of the vulvar masses revealed a helical, retractable, tubular structure with dense fluid content of low flow, which varied in size with pressure and had a morphology of nervous, varicose dysplasia. Varicography and ascending venography were performed by femoral approach to assess communications between varicosities and deep pelvic veins. No areas of arteriovenous dysplasia or reflux were observed on the tributaries of either the ovarian or the internal iliac veins. The examination was completed by catheterization of the vulvar mass on the right side. The vascular lake was delineated in the area of the right labium minus, which drained through estuarine tributaries into the iliac vein. The aforementioned findings were confirmed by magnetic resonance angiography (MRA), as shown in Figure 2. Vulvar varices of maximal diameter of 3 mm were reported, which were drained into dilated pelvic floor tributaries of the internal iliac vein. Connections with the long saphenous veins were also apparent through the superficial external pudendal vein; however, incompetence or dilatations were not noted. However, the supine position of the adolescent during the examination might have underestimated the degree of the disorder.

Due to the absence of chronic pelvic pain suggestive of Pelvic Congestion Syndrome (PCS), no intervention was decided for the adolescent, apart from lifestyle modification to ameliorate obesity health hazards. During 2-year follow-up visits, she remained asymptomatic and did not report any embarrassment or other negative feelings about the appearance of her vulvar varicosities, which neither enlarged nor reduced. She still had morbid obesity along with menstrual irregularities, and she was diagnosed with polycystic ovary syndrome.

## 3. Discussion

The first report of vulvar varices and their management was in 1967, though its affined Pelvic Congestion Syndrome (PCS) was known since the 19th century [10,11]. Thorough literature search did not reveal large studies addressing the diagnosis and treatment of vulvar varices, only cases reports and small case series. The rarity of the condition, especially in the nonpregnant state, has not allowed the development of definite diagnostic criteria and evidence-based treatment.

Vascular drainage of the vulvar area is achieved through external pudendal veins (superficial and deep), which drain into the long saphenous vein at the saphenous opening. Ιnternal pudendal veins are the second pathway of drainage of the labia and clitoris, venae comitantes of the internal pudendal artery, and tributaries of the internal iliac vein [12]. The third drainage pathway is by the vein of the round ligament running through the inguinal canal to drain to the ovarian vein [10].

Vulvar varices pathogenesis is poorly understood, because of the complexity and variation of the pelvic circulation. Sometimes the problem is isolated in the vulvar veins, whereas, when it is part of PCS, anastomoses and drainage in both great saphenous and internal iliac impede the discovery of a single affected proximal vessel. Adipose tissue participates in the pathogenesis of pelvic venous syndromes. Lower extremity varicosities are more frequent at higher BMI, whereas PCS is less frequent [7,13]. A possible explanation is that excess adipose tissue in the abdominal cavity causes increasing reflux on distal tributaries of pelvic veins and long saphenous vein; however, elastic properties of the adipose tissue protect deep pelvic veins from compression [6,8]. Additionally, it is proposed that the adipose tissue actively involves the renin–angiotensin system and, subsequently, controls the vascular tone [6,14].

The differential diagnosis of a vulvar mass in the nonpregnant state can be confusing. Bartholin gland disorders (cysts or abscesses) are the most probable diagnosis when a vulvar mass appears suddenly. Other type of cysts, dermoid or epidermoid, can also appear as nodules with variable characteristics on external genitalia. Inguinal or femoral hernias should also be excluded, especially when the mass increases in size along with intrabdominal pressure. Traumatic event can cause a hematoma, with transient appearance as nodule before creating a flat pigmented lesion. Vascular malformations, venous or arteriovenous, and hemangiomas usually present as bluish masses and make difficult the differential diagnosis without any imaging studies.

In most studies, work-up begins with doppler ultrasonography of pelvic veins to identify the affected vessels and valvular incompetence and measure the diameter of the dilated vessel and blood flow [3]. According to the American Venous Forum and Society for Vascular Surgery guidelines, patients with symptoms indicative of PCS should additionally undergo contrast venography by jugular or femoral vein catheter [15,16,17]. Varicography, which consists of direct injection of contrast into the visible varicose veins, provides additional help only in the pre-operative planning [18]. Computed tomography and magnetic resonance angiography can diagnose any associated PCS and identify the affected veins, which can be targeted for therapeutic interventions [16].

The choice of therapeutic approach in nonpregnant women is equivocal. Since there are not any randomized controlled studies, the investigation and treatment plan are usually individualized based on small-scale case series and case reports. The two main factors that guide the decision for intervention are symptomatology and association with PCS or leg varicosities. It is important with any venous circulation disorders that have been identified with imaging to design the treatment plan. Advanced imaging of the surrounding venous anatomy of the pelvis and legs should be obtained to guide management, which includes compression, sclerotherapy, embolization, or surgical ligation [19]. When vulvar varices are part of PCS, the affected ovarian or internal iliac vein is the target of the approach. When proximal venous reflux is treated, varices are usually reduced in size [4,20]. Minimally invasive techniques, like embolization and laparoscopic ligation of affected ovarian veins, are commonly used [3,4,20,21,22,23,24]. In cases of persistent varices or when there is no pathology in proximal pelvic circulation, vulvar sclerotherapy or excision of the varices can be performed. Gavrilov, in a case series of 61 nonpregnant patients, used as selection criteria for sclerotherapy venous diameter less than 6 mm and no connection between the varicose vein and main branch of internal iliac vein in doppler ultrasound or CT [3]. Both phlebectomy and sclerotherapy provide significant improvement in vulvar venous disorders, and the reported relapses are rare [3,25].

Since our adolescent did not report any symptoms and was not concerned about the aesthetics of her external genitalia, she was managed conservatively. The lack of cases of vulvar varicosities in the literature in adolescent nulliparous girls and the fact that adolescence is a period of body development guided our decision. Moreover, weight gain, in our case, seemed to coincide with the appearance of the varicosities, and, since this was a modifiable factor, lifestyle modification and weight reduction were advised before any therapeutic approach. In the current literature, there are only two other case reports of nulliparous young women with vulvar venous varicosities [4,5]. Leung et al. presented a case of a young 26-year-old nulliparous woman with a symptomatic large vulvar varicose vein and concurrent dilated right ovarian vein. Her past medical history revealed progressive enlargement of her right vulval swelling over 6 years with perineal heaviness and pain, which were exacerbated after prolonged standing. Ligation of the right ovarian vein laparoscopically improved the vulvar varices [4]. Naous et al. presented a 16-year-old adolescent with a minimal protruding right vulvar mass, which was reported to enlarge over 9 months and cause discomfort. Guided sclerotherapy was performed and resulted in reduction of the vulvar varicosity [5]. 

Relatively recent commendations from a multidisciplinary research consensus panel recognized the multiple evidence gaps on the spectrum of symptoms and signs related to the pelvic venous circulation and suggested that the term “syndrome” should be abandoned, and the term “pelvic venous disorders” should be used instead to avoid confusion. 

The panel acknowledged the need for multidisciplinary research to avoid over- or undertreatment of such cases [26]. 

Despite extensive imaging to exclude other pathologies, especially vascular malformations or hemangiomas, a conservative follow-up approach was considered appropriate in this asymptomatic adolescent case. Female sex, along with obesity, are known risk factors for varicose vein formation; however, the pathogenesis is not fully understood [27]. The role of excess adipose tissue in the pathophysiology of adolescent vulvar varicose veins and their optimal management remain to be elucidated.

## Data Availability

The datasets used in this study are available from the corresponding author.
